# Novel strategy for subretinal delivery in *Xenopus*

**Published:** 2011-11-16

**Authors:** Federico Gonzalez-Fernandez, Cheryl A. Dann, Mary Alice Garlipp

**Affiliations:** 1 Ross Eye Institute and Department of Ophthalmology, State University of New York, SUNY Eye Institute, Buffalo, NY; 2 Pathology & Anatomic Sciences; State University of New York, Buffalo, NY; 3 Graduate Program in Neurosciences; State University of New York, Buffalo, NY; 4 Veterinary Medical Unit, Medical Research Service, Veterans Affairs Medical Center, Buffalo, New York, Buffalo, NY; 5 Medical Research Service, and Departments of Ophthalmology and Pathology, Veterans Affairs Medical Center, Buffalo, NY

## Abstract

**Purpose:**

The subretinal space, which borders the retinal pigment epithelium (RPE), photoreceptors, and Müller cells, is an ideal location to deliver genetic vectors, morpholino oligos, and nanopharmaceuticals. Unfortunately, materials injected into the space tend to stay localized, and degenerative changes secondary to retinal detachment limit its usefulness. Furthermore, such injection requires penetration of the sclera, RPE/choroid, or the retina itself. Here, we developed a strategy in *Xenopus* to utilize the continuity of the brain ventricle and optic vesicle lumen during embryogenesis as a means to access the subretinal space.

**Methods:**

Wild-type and transgenic embryos expressing green fluorescent protein under the rod-opsin promoter were used for optic vesicle and brain ventricle injections. For injection directly into the optic vesicle, embryos were laid on one side in clay troughs. For brain ventricle injections, embryos were placed standing in foxholes cored from agarose dishes. Linear arrays with each embryo positioned dorsal side toward the micromanipulator facilitated high throughput injections. Twenty-five micrometer micropipettes, which were positioned with a micromanipulator or by hand, were used to pressure inject ~1.0 nl of test solution (brilliant blue, India ink, fluorescein isothiocyanate dextran, or 0.04 µm of latex polystyrene microspheres [FluoSpheres^®^]). FluroSpheres® were particularly useful in confirming successful injections in living embryos. Anesthetized embryos and tadpoles were fixed in 4% paraformaldehyde and cryoprotected for frozen sections, or dehydrated in ethanol and embedded in methacrylate resin compatible with the microspheres.

**Results:**

Direct optic vesicle injections resulted in filling of the brain ventricle, contralateral optic vesicle, and central canal. Stages 24 and 25 gave the most consistent results. However, even with experience, the success rate was only ~25%. Targeting the vesicle was even more difficult beyond stage 26 due to the flattening of the lumen. In contrast, brain ventricle injections were easier to perform and had a ~90% success rate. The most consistent results were obtained in targeting the diencephalic ventricle, which is located along the midline, and protrudes anteriorly just under the frontal ectoderm and prosencephalon. An anterior midline approach conveniently accessed the ventricle without disturbing the optic vesicles. Beyond stage 30, optic vesicle filling did not occur, presumably due to closure of the connection between the ventricular system and the optic vesicles. Securing the embryos in an upright position in the agarose foxholes allowed convenient access to the frontal cephalic region. On methacrylate sections, the RPE-neural retina interphase was intact and labeled with the microspheres. As development continued, no distortion or malformation of the orbital structures was detected. In green fluorescent protein (GFP), transgenic embryos allowed to develop to stage 41, retinal FluoSpheres^®^ labeling and photoreceptor GFP expression could be observed through the pupil. On cryosections, it was found that the FluoSpheres^®^ extended from the diencephalon along the embryonic optic nerve to the ventral subretinal area. GFP expression was restricted to rod photoreceptors. The microspheres were restricted to the subretinal region, except focally at the lip of the optic cup, where they were present within the retina; this was presumably due to incomplete formation of the peripheral zonulae adherens. Embryos showed normal anatomic relationships, and formation of eye and lens appeared to take place normally with lamination of the retina into its ganglion cell and the inner and outer nuclear layers.

**Conclusions:**

Diencephalic ventricular injection before stage 31 provides an efficient strategy to introduce molecules into the embryonic *Xenopus* subretinal space with minimal to the developing eye or retina.

## Introduction

The vertebrate eye arises through a series of reciprocal inductive interactions between the neuroepithelium, surface ectoderm, and extraocular mesenchyme. Central to this choreography is the formation of the optic cup through the invagination of the optic vesicle. As the vesicle induces lens formation in the overlying competent surface ectoderm, its inner layer in turn gives rise to the neural retina, while the outer layer becomes the retinal pigment epithelium (RPE). As this is taking place, the optic stalk narrows, eventually separating the central nervous system (CNS) ventricles and subretinal space into unique compartments [1,2]. With the elongation of the outer segments, the interphotoreceptor matrix (IPM) accumulates within the expanding subretinal space. RPE zonula occludens prevent diffusion of matrix components sclera; while the zonula adherens prevents molecules with a Stokes’ radius >30 Å from leaving the subretinal space vitread between adjacent photoreceptor and Müller cells [3]. The matrix is thought to mediate RPE/retina interactions during development, including adhesion, sequestration of growth factors, and facilitating the exchange of retinoids between the RPE and neural retina in the visual cycle [4-8].

Because it borders the RPE, photoreceptors, and Müller cells, the subretinal space is an ideal location for delivering molecules to the outer retina. Subretinal injection can be easily performed in rodents [9-11], and has been useful for the introduction of viral vectors [12-14] and growth factors into the retina [15-18]. Many of these studies have been extended into clinical trials, further establishing the usefulness of the subretinal space in gene delivery [13,19-21].

Although the IPM is an ideal location to deliver molecules to the retina, several factors limit the usefulness of subretinal injections. In theory, material injected into the subretinal space should have access to the entire matrix, and therefore all of the RPE, photoreceptors, and Müller cells. However, injected material tends to stay localized at the injection site [9,10]. Furthermore, pathological changes secondary to the injection itself are not uncommon, as the method requires penetration of the sclera and RPE/choroid, or the retina itself. Finally, retinal detachment, an unavoidable consequence of subretinal injection, can alter ocular gene expression, and result in retinal and RPE degeneration [22,23].

A method allowing the less traumatic introduction of molecules into the subretinal space would be very useful for basic and translational research. In particular, *Xenopus*, a favorite of the experimental embryologist, develops exogenously, can be manipulated surgically, and is amendable for transgenic strategies. However, the inability to deliver test compounds to the retina and subretinal space remains a major obstacle. For example, it would be useful to be able to deliver DNA constructs, morpholino oligos, and morphogens into the subretinal space to study photoreceptor gene expression and function. Currently, morpholinos are commonly introduced into the embryo by blastula injection. However, when injected in this way, the morpholino is diluted out during organogenesis, limiting its ability to inhibit mRNA translation at late stages such as those needed to study photoreceptor development and function.

**Figure 1:**
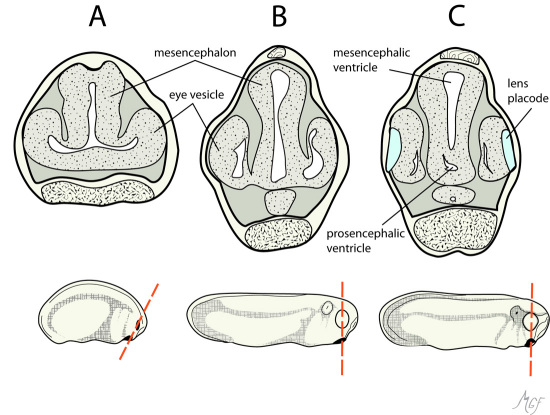
The relationship between the optic vesicle and brain ventricles in the developing *Xenopus* embryo are shown in these diagrams. The cross-sections, which are through the optic vesicles (dashed lines), compare stages 23, 26, and 27 in **A, B**, and **C** respectively. These drawings, were prepared based on [24,30], to illustrate that the brain ventricle and optic vesicle lumens are continuous at least at stage 23.

To circumvent a direct injection approach, we wanted to develop a less invasive strategy to deliver molecules to the subretinal space without disruption of the retina. Such a method should not disturb the retina anatomically. We considered that the normal developmental sequence of the CNS and ocular structures could provide an opportunity to accomplish this goal. Figure 1 illustrates the lumen of the optic vesicle is continuous with that of the brain ventricle early in development [24]. We anticipated that material introduced into the optic vesicle or brain ventricle would become sequestered within the subretinal space as the eye forms. Our studies suggest that optic vesicle or brain ventricle microinjection provides a nondisruptive strategy to deliver molecules into the subretinal space.

## Methods

This research was performed under protocols approved by the Veterans Affairs Medical Center Institutional Animal Care and Use Committee. Adult pigmented *Xenopus laevis* were obtained from *Xenopus* 1 (Dexter, MI) or NASCO (Fort Atkinson, WI). Animals were maintained under a 12 h:12 h light-dark cycle in an XR1 aquarium system (Aquatic Habitats, Apopka, FL). Lines of transgenic animals expressing GFP under the rod-opsin promoter [25] were used in some experiments to identify the developing photoreceptors. These animals were generated by restriction enzyme mediated integration [26,27], using linearized plasmid pXO-GFP generously provided by Dr. Barry Knox (SUNY, Syracuse, NY) [28]. Methods for embryo care and *Xenopus* husbandry were followed as previously described [29]. Embryos were staged according to Nieuwkoop and Faber [30]. The following describes embryo handling, the subretinal delivery method, and useful photography and histological notes.

### Embryo preparation

Embryos for injection can be obtained either by natural or in vitro fertilization. Although the latter has the advantage of generating embryos at nearly identical developmental stages, natural fertilization is often convenient, especially when different stages are desired. The preference therefore largely lies in what is routinely available in the laboratory. For either method, it is helpful to maintain groups of developing embryos at various temperatures ranging from 14 to 22 °C. This lengthens the time window when the desired stages are available. Khokha et al. [31] provides charts of the effect of temperature on *Xenopus laevis* and *Xenopus tropicalis* development (see also Xenopus development).

Embryos are secured for microinjections in modeling clay or agarose dishes, and for most routine work, visualized under Stemi 2000 stereomicroscopes equipped with zoom magnification (Carl Zeiss, Germany). Although embryos at the developmental stages used for injection are not free swimming, spontaneous movements can interfere with securing the animals and performing injections. Benzocaine (Sigma; St Louis, MO), at a final concentration of 0.005 to 0.01%, is effective in eliminating such movements (see comments under troubleshooting guide, Table 1). For injections directly into the optic vesicle, the embryo is laid on its side in a trough formed in modeling clay. Having tried several types of modeling clay, we found that the blue nonhardening/nontoxic type such as that from Sargent Art (Hazleton, PA) has the right malleability without any apparent toxicity to the embryos. The embryo is positioned in the clay as described below, and a flap of clay gently brought over to secure it in place.

**Table 1:** Trouble shooting guide

**Category**	**Problem**	**Recommendation**
Embryo handling	Embryos damaged during insertion into the agarose foxholes.	Add 0.05% food coloring to the agarose to improve visualization of the holes. Tip: touch closed forceps to cement gland, and drag embryo over hole. The gland will adhere lightly to the forceps. Then, push embryo backward guiding its posterior end into the hole with a second forceps (not illustrated in video)
	Spontaneous movements interfere with foxhole insertion and microinjection.	Add 0.01% benzocaine to bath (a 0.05% stock keeps for at least one month at 4 °C). (Note: MS-222, a commonly used fish and amphibian anesthetic, impairs visual pigment regeneration by binding 11-*cis* retinal [40], which presumably is responsible for its toxicity [41]. For these reasons, we prefer benzocaine.
	Embryos damaged during removal from foxholes.	Embryos are extremely delicate! Never grab. Once in the hole, the strength of the cement gland adhesion is insufficient to pull the embryo out. The best method is to use a large bore plastic, or fire-polished glass pipette. Position the pipette over the hole lightly touching the agarose and apply gentle suction. The embryo will readily pop out, and the hole can be reused for another embryo.
	Uncertain that material is actually being injected.	Addition of colored or fluorescent dye is helpful in confirming the injection. 0.025 - 0.05% phenol red is convenient. Alternatively fluorescent dextran or beads can be used. Successful injections can be confirmed by visualizing the embryo in vivo under a fluorescent stereomicroscope, and in histological sections. Adjust the backpressure so that a gentle flow of material continually emerges from the tip. If the backpressure is insufficient, water will flow into the pipette diluting the material intended for injection. Discard embryos that do not show typical pattern of ventricular/optic vesicle filling (see Figure 3, Figure 6, and Figure 9).
	Insufficient number of embryos injected	The injection window is finite - stay organized! The day before, check that enough nitrogen gas and micropipettes are available. Set up multiple injection stations. Rear embryos at various reduced temperatures (14 – 20 °C) to widen window of specific stage availability. Alternatively, carry out in vitro fertilizations at several points during the day.
Micropipette problems	Difficulty filling pipette from limited supply of valuable injection material.	Under a stereomicroscope lower by hand, or with the aide of a micromanipulator, the micropipette tip almost horizontally into a droplet on a Parafilm rectangle. While carefully ensuring that the tip remains within the drop, start suction. Do not remove the tip until the vacuum has finished.
	Difficulty in positioning micropipette	A micromanipulator can be helpful. However, we find that it is more efficient to hold the micropipette directly by its handle. Rest fingers on microscope stage, and keep the glass pipette short (~1 cm) to minimize hand vibrations.
Injections Photography and Tissue Processing	No ventricle or optic vesicle fill observed. Optic vesicle not filling despite brain ventricle filling. Injected material not confined to cerebral ventricles, or optic vesicle. Poor contrast of photographed embryos Spontaneous drifting of embryo during photography Fixation and sectioning are laborious and time consuming Fluorescence not visible in sections cut from paraffin embedded embryos.	Generally due to tip obstruction. Clogs occur from tissue or agarose stuck in the tip and/or drying of test solution when pipette is taken out of the bath for example between dishes. The tip can be broken to restart the flow. Avoid leaving the pipette out of water for any extended time. A gentle dye flow from the pipette tip is a good sign that the tip is not clogged. Separation of optic vesicle and neural tube lumens may have occurred. Use younger embryos (< stage 31). Penetration too deep, or injection pressure too high. Note that the diencephalic ventricle is just under the frontal ectoderm (see Figure 3). To enter the ventricle, it is therefore only necessary to puncture through the thin ectoderm and proencephalon. Do not overfill. Find the maximum pressure and time settings that do not cause visible inflation of the optic vesicles during the injection. Use fiber optic light source angled ~30 ° from one side. Living embryos drift forward. One solution is to formalin fix before photographing. For living embryos, lay broken coverslip pieces in front of embryo to prevent movement. Tissue processing and sectioning of multiple embryos is often the most challenging part of any experiment. Fixation and sucrose/OCT infiltration are efficiently performed in 24-well cell culture plates. To orient multiple embryos so that they can be simultaneously sectioned through their eyes, align four to six embryos lying down in the embedding mold so that their anterior ends all touch the side of mold. Move the embryos within the viscous OCT with a metal needle. Taking care not to disturb their alignment, freeze them in an isopentane bath over liquid nitrogen. Working quickly cut out the aligned embryos in a single rectangular block. Re-embed the block in a heads down orientation in a fresh mold prefilled with OCT and freeze. In this way, the alignment is exact, allowing the eyes from all embryos to be included in same histological section. The xylene treatment required for tissue infiltration with paraffin is not compatible with the Cy3-FluoSpheres®. Embed in methacrylate resin (Figure 7), or perform frozen sections (Figure 11).

**Figure 2:**
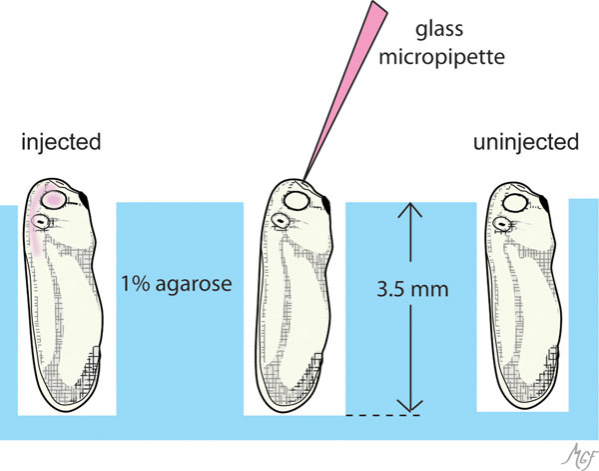
This diagram illustrates the position of embryos in agarose dishes for brain ventricle injections. Embryos were placed in foxhole arrays punched out of 1% agarose dishes (blue). At least 25 foxholes can be prepared per dish. The holes are made to a depth so that the head protrudes slightly out of the foxhole (see Methods). The embryonic diencephalic ventricle is located along the midline, and protrudes anteriorly just under the frontal ectoderm and prosencephalon (compare with Figure 3). A midline anterior approach conveniently accesses the ventricle without disturbing the optic vesicles (middle embryo). The injected material (pink) readily fills the brain ventricle with optic vesicle, and typically can be appreciated extending into the central canal [30].

For brain ventricle injection, we found it was best to orient the embryo in a vertical “standing position” in 1% agarose (BioRad; Hercules, CA) dishes as shown in Figure 2. We adapted a method previously described to orient zebrafish embryos for brain ventricle injections [32]. In adapting the method for *Xenopus*, we considered that the overall length of a stage 28 embryo is approximately 4 mm. For 60×15 mm plastic Petri dishes with a base diameter of 5.0 cm, 6.9 ml of agarose gives a thickness of ~3.5 mm allowing the head to protrude slightly out of the foxhole. The holes are punched out under water using a Pasteur pipette broken 1.0 cm from its tip (5 3/4” flint glass, Cat#13–678–6A; Fisher). This gives a final hole diameter of ~1.25 mm, which accommodates the embryo well. It is best to arrange the holes in linear arrays, and position each embryo with the dorsal side toward the micromanipulator. This facilitates performing numerous injections and keeping track of the injected embryos.

A generally useful trick for gently positioning embryos without directly handling them is to make use of their cement gland, an anterior-ventral pigmented structure. Embryos use this mucin-secreting organ to attach to stationary supports before being able to swim. The cement gland also appears to have a sensory function, stopping the embryo from moving once attached [33]. Embryos will stick to the forceps tip at the cement gland, and can then be dragged up and over the foxholes. The embryo is then pushed backward into the hole while guiding the tail end downward with a second forceps.

### Subretinal delivery methods

**Figure 3:**
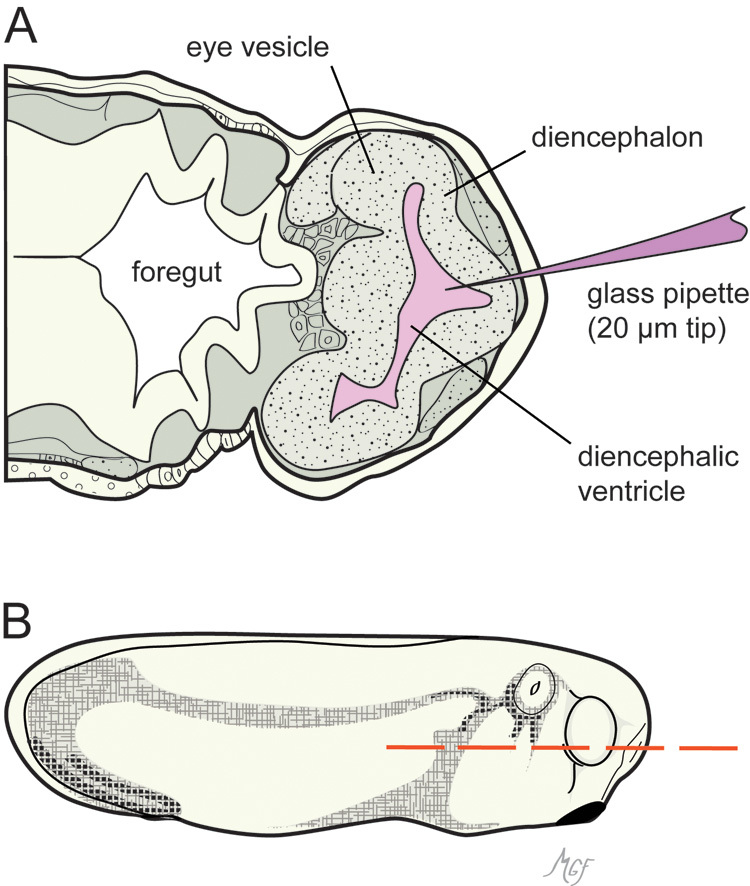
Horizontal section of a stage 26 embryo illustrating the brain ventricle injection method used to introduce materials into the optic vesicle lumen. **A**: A glass micropipette was introduced into the diencephalic ventricle anteriorly. **B**: The dashed line shows the orientation of the section in panel **A**. The drawings were prepared based on [24,30].

**Figure 4:**
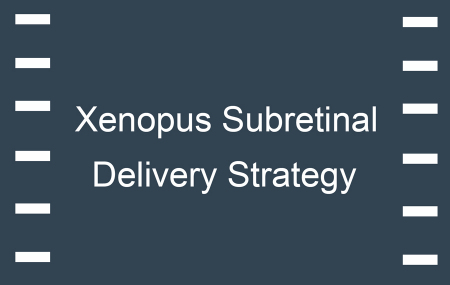
*Xenopus* subretinal delivery strategy. Animation 1. The attached movie illustrates the diencephalic injection strategy to introduce microspheres into the optic vesicle of *Xenopus laevis* embryos. Note that the slide bar at the bottom of the quicktime movie can be used to manually control the flow of the movie. If you are unable to view the movie, a representative frame is included.

Subretinal delivery can be accomplished by direct optic vesicle or brain ventricle injection. Securing the embryos for injection using either method is described above. For optic vesicle injection, embryos were tilted 30° to one side in clay dishes; for brain ventricle injections, the embryos were positioned vertically in agarose dish foxholes (Figure 2 and Figure 3). For both types of injection, glass micropipettes (1BBL no fill 1.0mm 4 in. cat# 1B100–4; World Precision Instruments, Sarasota, FL) were prepared on a micropipette puller. Tips were carefully broken by hand using jeweler’s forceps to a 20 to 30 µm outer diameter. The micropipettes were positioned with a Manual LH mechanical micromanipulator (Harvard Apparatus; Holliston, MA). We found that particularly for the brain ventricle injections, the micropipette could be positioned equally well by hand. This was more efficient when injecting numerous embryos (>25 embryos per dish). A PLI-100 microinjection system (Harvard Apparatus) was used to pressure inject approximately 10.0 nl of test solution to either the optic vesicle or brain ventricle. Typically, pressure injection per se was not necessary, and filling was accomplished simply by the system’s backpressure. Diluted blue food coloring (brilliant blue) was useful to help visualize the injection. The injected dye can be visualized accumulating in the optic vesicle lumen or brain ventricle under the ectoderm. In the last injection series of the included movie (Figure 4), the darkening of the optic vesicle represents such filling. Extension into the neural canal could often be observed. Materials injected included India ink, fluorescein isothiocyanate (FITC) dextran, and fluorescent 0.04 µm latex-polystyrene microspheres (FluoSpheres^®^, Life Technologies, Carslbad, CA). Cy3-FluoSpheres^®^ were used to visualize injections into living embryos using a Stemi SV 11 epifluorescence microscope with an HBO103 mercury lamp and 29004 ET-Cy3 filter cube (ET545/25×, T565lpxr, ET605/70m; Chroma, Bellows Fall, VT). In contrast to the developing mammalian retina, *Xenopus* embryonic tissues, including the retina, contain autofluorescent cytoplasmic yoke granules. Cy3 and FITC dyes were selected for the injection markers, as they could be clearly distinguished from the tissue autofluorescence while allowing the possibility of using additional fluorochromes emitting further in the red for immunofluorescence double labeling. The reader is referred to additional helpful published protocols on the subject [34,35].

### Histology and microscopy

For imaging the FluoSpheres^®^ injection in vivo, living embryos were visualized with a Zeiss Stemi 11 fluorescence stereomicroscope. For histological evaluation, anesthetized embryos and tadpoles were fixed in 4% paraformaldehyde and cryoprotected through graded sucrose solutions (final, 20% sucrose), followed by infiltration with Optimal Cutting Temperature compound (Ted Pella, Redding, CA; see notes in Table 1). Alternatively, fixed embryos were dehydrated in ethanol and embedded in Lowicryl resin according to the manufacturer (London Resin Co.). Lowicryl sections have excellent morphological detail, and being a methacrylate-based hydrophilic resin, Lowicryl is compatible with the latex-polystyrene FluoSpheres^®^. Confocal microscopy was performed on an Axiovert 200 motorized microscope equipped with a Zeiss LSM 510 system (laser lines: GFP, ex 477 nm; Cy3, ex 565 nm). The multiphoton capability used to visualize 4',6-diamidino-2-phenylindole (DAPI) was provided through a Coherent Chameleon Ultra II laser. Data collection and image analysis were performed with LSM software version 4.2, and figures created in Adobe Creative Suite version 4.0 (San Jose, CA).

## Results

**Figure 5:**
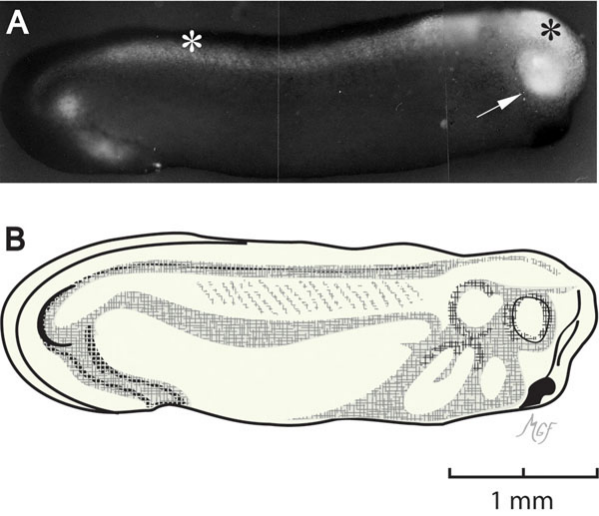
Optic vesicle fluorescein isothiocyanate–dextran injection. The injection was performed at stage 24, and photographed here at stage 28. **A**: Fluorescence shows filling of the optic vesicle (arrow), brain ventricle (black asterisk), and central canal (white asterisk). **B**: This illustration was prepared to be consistent with stage 28 as in reference [30].

We compared the effect of embryonic stage and injection route on the ability to target the subretinal space. In our initial experiments, we injected the optic vesicle directly at stages 24 through 26. The most consistent fills were obtained at stages 24 and 25, where the optic vesicle lumen appears to be at its largest volume. Targeting the vesicle is more difficult beyond stage 26 due to flattening of the lumen. We positioned the micropipette on the transverse plane near the apex of the protruding vesicle. Filling of the injected vesicle with FITC dextran was almost immediate, followed by the brain ventricle and contralateral optic vesicle (Figure 5). After one to two minutes, the label diffused into the central canal (white asterisk). Compared to dextran, the fluorescent microspheres showed less rapid canal diffusion, but readily filled the optic vesicles and brain ventricle (see below).

Our second strategy took advantage of the continuity of the brain ventricle with the lumen of the embryonic optic vesicle. Although the ventricle lumen is accessible from a variety of injection angles, we found that an anterior midline approach gave the most consistent fills. As shown in Figure 3A, the embryonic diencephalic ventricle is located along the midline, and protrudes anteriorly just under the frontal ectoderm. A midline anterior approach conveniently accesses the ventricle without disturbing the optic vesicles. Securing the embryos in an upright vertical position in agarose dishes allowed convenient access to the frontal cephalic region, and facilitated the injection of multiple embryos (Figure 2).

**Figure 6:**
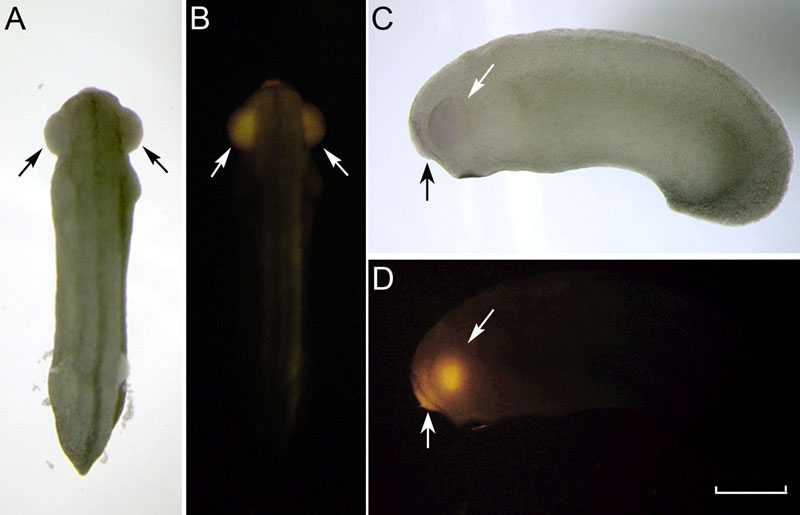
Cy3 microspheres were injected into the diencephalic ventricle at stage 26. Panels **A**, **B** and **C**, **D** are dorsal and lateral views, respectively, of the same embryo showing the optic vesicles, which normally appear to bulge laterally from the head (slanted arrows). Under fluorescence, the optic vesicles are filled with FluoSpheres^®^ (panels **B**, **D**). The vertical arrow in panels **C**, **D** indicates the diencephalic ventricle which also contains the FluoSpheres^®^. The scale bar in the lower right equals 0.5 mm.

**Figure 7:**
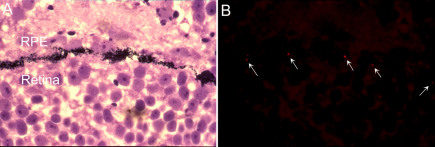
Texas red microsphere were delivered to retinal pigment epithelium/retina interface. The contralateral optic vesicle was injected with the microspheres at stage 26. **A**: At stage 28, the embryo was embedded in methacrylate polymer. At this stage, formation of the optic cup is complete and melanin pigment is visible in the retinal pigment epithelium. **B**: The same section photographed under fluorescence shows the microspheres appearing as red dots (arrows) along the RPE/retina interface.

**Figure 8:**
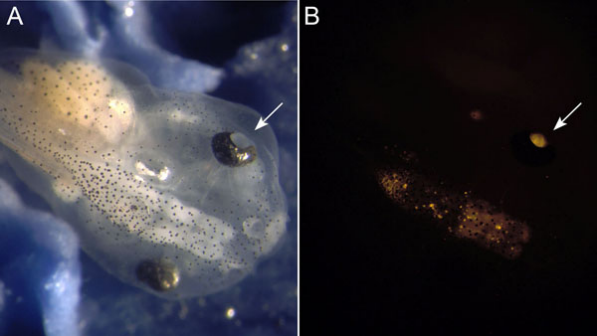
FluoSphere^®^ labeling in the tadpole eye. Four days prior, this living, free-swimming tadpole received a Cy3-FluoSpheres^®^ brain ventricle injection at stage 26. **A**: Macroscopically, there is no apparent distortion or malformation of the orbital structures. **B**: Under fluorescence, the label is restricted to the brain and retina. In the eye, the fluorescence emanates through the pupil (arrow). Although the right eye was equally labeled, its fluorescence is not visible, as its pupil is not in view.

We anticipated that material introduced into the diencephalic ventricle would diffuse into the optic vesicles bilaterally; vesicle involution would sequester the material into embryonic subretinal space. The distribution of fluorescent microspheres after stage 26 injections are shown in a living embryo (Figure 6) ~15 min after the injection, and histologically (Figure 7) following optic cup formation (~stage 28). Figure 6A,C shows dorsal and lateral views, respectively, illustrating the optic vesicles, which normally appear to bulge from the head laterally (slanted arrows). Viewed dorsally under fluorescence, filling of both vesicles can be seen; accordingly, when viewed laterally, filling of the diencephalic ventricle can also be observed (vertical arrow). In Figure 7, formation of the optic cup is complete, and the outer layer of the cup has become pigmented, which is indicative of RPE differentiation (Figure 7A). The outer retina shows little evidence of differentiation such as retinal lamination or outer segment formation. The tissue in this photomicrograph was embedded in methacrylate, a hydrophilic resin, which provides higher resolution than paraffin or frozen sections, and is compatible with the FluoSpheres^®^ (organic solvents such as xylenes required for wax infiltration compromise the integrity of the microspheres). When viewed under fluorescence (Figure 7B), microspheres injected in the contralateral vesicle are found distributed at the interphase between the RPE and neural retina. Whether the microspheres are present in the interphotoreceptor matrix or in the cytoplasm of cells bordering the subretinal space cannot be determined from this photomicrograph. As the embryo continues to develop into a free-swimming tadpole, we do not detect any distortion or malformation of the orbital structures (Figure 8A), and retinal labeling appears as fluorescence emanating through the pupil (arrow in Figure 8B). These findings support the conclusion that subretinal access via brain ventricle injection or injection of the contralateral optic vesicle does not disturb the normal formation of the ocular structures, nor does it prevent the normal involution of the vesicle and apposition of the RPE with the neural retina.

**Figure 9:**
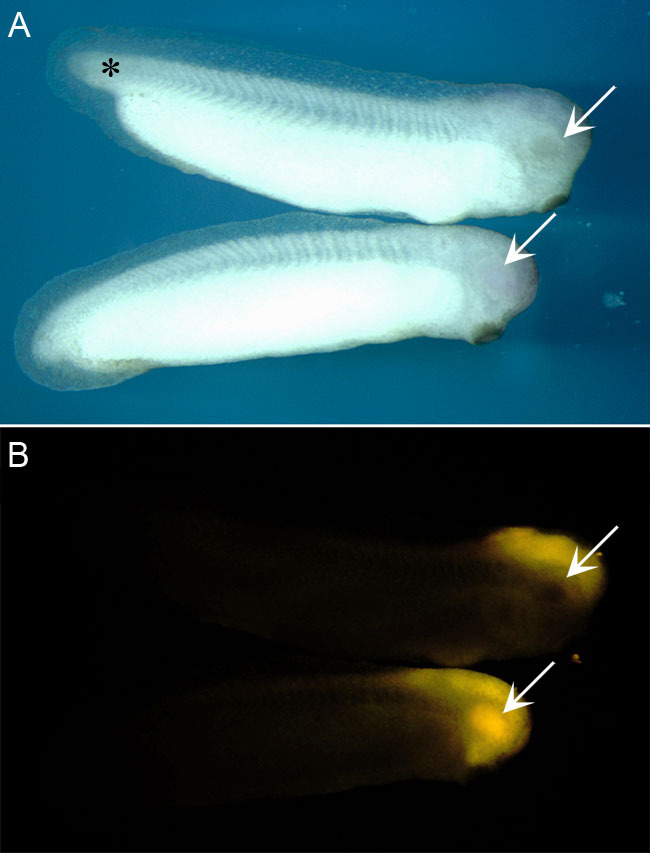
Cy3-FluoSpheres^®^ were injectedbefore, and after separation of the optic vesicle lumen from the brain ventricular system. These photographs were taken 10 min after injection into the diencephalic ventricle. **A**: The older stage 31 (upper) embryo has a more advanced overall body shape, with fuller development of its tail bud (*) compared to the stage 29 embryo beneath it. The arrows show the optic vesicles. **B**: Under fluorescence, although brain ventricle labeling is seen in both embryos, optic vesicle filling is seen only in the stage 29 embryo (arrows).

**Figure 10:**
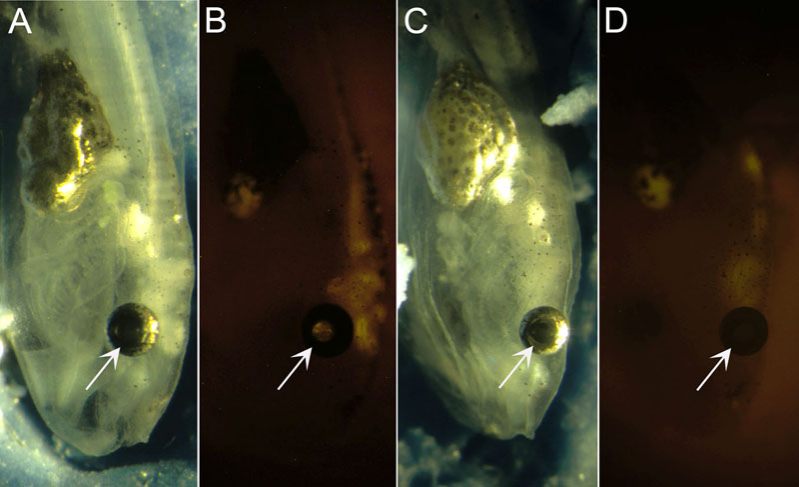
Retina access is dependent on the embryonic stage. The diencephalic ventricles were injected at stage 24 (**A**, **B**) or stage 31 (**C**, **D**), and the embryos allowed to mature to swimming tadpoles. The eyes of both tadpoles appear to have developed normally. Cy3 fluorescence, which is appreciated through the pupil (arrow), is present only in the stage 24 injected tadpole. Such retinal labeling can be achieved through stage 29/30. For stages 31 and older, brain ventricle injection does not support delivery to the retina.

Access to the optic vesicle via the brain ventricle was dependent on the embryonic stage. Figure 9 compares embryos at stages 29 and 31 photographed immediately after injection. Here, diencephalic ventricular injection supported optic vesicle filling at stage 29, but not at stage 31 (Figure 9B). The two embryos in Figure 10 were injected either at stage 24 or 31, and allowed to mature to swimming tadpoles. Although the eyes of both tadpoles developed normally, fluorescence was detected only in the tadpole injected at stage 24. These results suggest that the connection between the brain ventricle and optic vesicle closes beyond stage 30.

**Figure 11:**
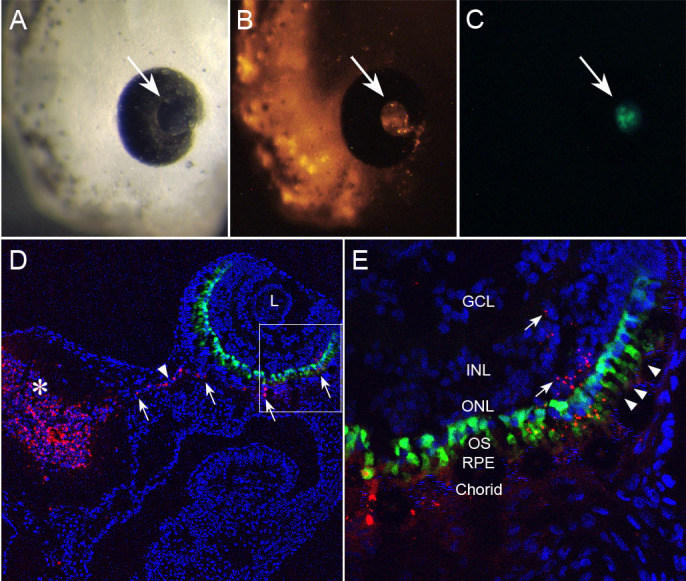
Diencephalic ventricular injection was used to deliver Cy3-FluoSpheres^®^ to the retina of transgenic tadpoles expressing green fluorescent protein under the rod-opsin promoter. The brain ventricle was injected through the anterior prosencephalon at embryonic stage 27, and development allowed to continue for 48 h to stage 41. **A**: In vivo photograph showing the normal external appearance of the eye and pupil (arrow). **B**: Cy3-FluoSphere^®^ labeling of the retina can be viewed through the pupil. **C**: Retinal expression of green fluorescent protein (GFP) is apparent through the pupil. **D**: Cryosection (transverse plane) through the eye at the level of the optic nerve (arrowhead) shows that the Cy3-FluoSpheres^®^ have diffusely labeled the diencephalon (asterisk), and are found extending through the optic nerve to the subretinal region. **E**: This higher magnification of boxed area in panel **D** shows GCL, ganglion cell layer; inner nuclear layer, ONL, outer nuclear layer; OS, outer segments; RPE, retinal pigmented epithelium; Arrowheads, rod photoreceptors expressing GFP.

In Figure 11, the diencephalic ventricle of a GFP-rod opsin transgenic embryo was injected through the anterior prosencephalon at stage 29. The embryo was then allowed to develop to stage 41. As in other embryos receiving such injections, the external appearance of the eye and pupil were unaffected (Figure 11A-C). FluoSphere^®^ labeling of the retina and photoreceptor GFP expression could be observed through the pupil. The cryosection shown in Figure 11D,E uses DAPI as a fluorescent nuclear counterstain. GFP expression is restricted to rod photoreceptors, which are still undergoing outer segment elongation (arrowheads, Figure 11E). The FluoSpheres^®^ labeled the entire diencephalon (asterisk, Figure 11D), presumably by direct diffusion from the ventricle lumen. In Figure 11D, the microspheres extend from the diencephalon to the eye along the embryonic optic nerve (arrowhead, Figure 11D). Higher magnification shows the FluoSpheres^®^ in the ventral subretinal area (Figure 11E). Peripherally, the microspheres are focally present within the outer and inner nuclear layers (arrows, Figure 11E). Overall, the embryo shows normal anatomic relationships of the diencephalon, eye, and gastrointestinal lumen. Furthermore, formation of the eye and lens appears to have taken place normally with lamination of the retina into its ganglion cell, and inner and outer nuclear layers. The above findings are consistent with the developmental stage, and suggest that the procedure does not significantly affect the formation of the eye and retina.

## Discussion

Direct access to the subretinal space in vivo is problematic due to the consequences of retinal detachment. However, in vitro systems do not always adequately model the complex interactions between the RPE, interphotoreceptor matrix, and neural retina. The motivation for the present study was to develop a strategy to introduce molecules into the subretinal space without disturbing the relationship between the RPE and neural retina. We reasoned that material injected into the common embryonic CNS lumen would diffuse to both optic vesicles. Subsequent involution of the vesicles would bring the material into the subretinal space of the optic cups. A particular advantage of this method is that the injected material has access to the entire RPE, photoreceptor layer, and Müller cell glia. In contrast, subretinal injections as typically performed generally allow access only to a limited portion of the interphotoreceptor matrix [9]. Previous studies in *Xenopus* using intraretinal injection and electroporation achieved only focal labeling of the retina [36]. Although the overall strategy is feasible, advantages and limitations of the method are presented so that it can be tailored for different experimental paradigms. With a little practice, the injections can be performed routinely even by those with little *Xenopus* experience.

We began by attempting to inject the optic vesicles directly. Direct injection into the optic vesicle could be advantageous when the opposite retina is needed as a control, or when brain injection is not desirable (for example, in optic nerve tracking studies). However, although it can be an effective way to deliver to the subretinal space of both retinas, the success rate was low (~25%) even with experience. Furthermore, one of the eyes was unavoidably subjected to the trauma of the injection itself. The main problem was accurately accessing the appropriate depth of penetration for the micropipette. It should be kept in mind that the vesicle lumen at most stages is a compressed flattened chamber, and therefore difficult to hit (note that the lateral protrusions of the optic vesicle are mainly due to the thickening of the developing inner neural retina). Ohnuma et al. [37] reported some experience using direct optic vesicle microinjections to transfect plasmid constructs in *Xenopus laevis*. Using stage 24 embryos, they found that the transfection efficiency for injections made “outside” the vesicle (lumen region) was only half that of injections made “inside” (between the neural retina and ectoderm). It is not known whether this regional difference reflects a biologic difference, or difficulties in targeting the optic vesicle lumen. Brain ventricle injection as an avenue to deliver material to the optic vesicles was not attempted in that study, or in any other study that we are aware of in the literature.

Brain ventricle injection had several advantages over direct optic vesicle injection. The most significant was that the diencephalic ventricle has a larger volume compared to that of the optic vesicle. This allows it to accept injections along a wider range of micropipette penetration depths. Ninety percent or more of the injections are successful. Furthermore, brain ventricle injection avoids subjecting an eye to the trauma of the injection. Using the brain ventricular injection, we have not observed any abnormality in the development of the ocular structures. The pupil, rate and extent of pigmentation, external appearance of the globe, histological lamination of the retina, and rod photoreceptor differentiation all appear to be indistinguishable from controls. Finally, the brain ventricle method lends itself well to a higher throughput compare to direct optic vesicle injections. Here, the use of agarose dish foxholes facilitates orienting numerous embryos for injection. Hundreds of embryos can be injected in a single morning. Finally, it should be pointed out that any experimental paradigm using the diencephalic ventricle injection or the direct optic vesicle route should include appropriate experimental controls. Such controls would be critical to distinguishing a genuine biologic phenotype from nonspecific effects of the injection, carrier, and/or the test molecule itself.

Several issues should be considered when planning experiments using the delivery method described here. A potential limitation of the method is that delivery may not be uniform throughout the subretinal compartment for all developmental stages. In the brain ventricle injection shown in Figure 11, labeling was restricted to the dorsal retinal space. This may represent a positional effect due to gravity, or a regional difference in the accessibility of the subretinal space. It should be pointed out that confinement of any material introduced into the subretinal space will depend on a variety of factors, including the rate at which the material leaves the space. Turnover of molecules introduced into the interphotoreceptor matrix could depend on the integrity of the external limiting membrane and the size of the molecule. The exclusion limit of the zonulae adherens in rabbits is between 30Å and 36Å [3]. For example, interphotoreceptor retinoid-binding protein (140 kDa), a normal component of the interphotoreceptor matrix, exceeds this exclusion limit, and is therefore confined to the subretinal space where it accumulates [4]. However, smaller molecules would not be excluded, and therefore should diffuse into the retina (this may be desired depending on the experimental objectives). The labeling of the peripheral retina (Figure 11E) may be due to incomplete formation of the zonulae adherens in this less-developed region of the retina. Thus, confinement to the subretinal space may be dependent on the stage at which the injection is performed. Ongoing experiments in our laboratory are addressing the above issues.

Brain ventricle injection as a way to deliver molecules or microspheres to the subretinal space can be applied only when the optic vesicle lumen is continuous with the CNS ventricular space. There is little information in the literature about the timing of the closure of the passageway. Detailed published anatomic drawings suggest that the passage between the compartments is lost from stage 26 onward [24]. In contrast, we found that FluoSpheres^®^ can diffuse from the diencephalic ventricle to the optic vesicle through stages 29/30. From stage 31 onward, the connection between the brain ventricle and subretinal compartment does not allow passage of the 0.04 µm microspheres. This does not rule out that the passage is patent to materials < 0.04 µm in diameter.

The subretinal delivery strategy described here may be applicable to some other systems where the subretinal space forms through the involution of the optic vesicle. In its present form, the method could probably not be applied to most invertebrates due to the absence of a common shared chamber similar to the vertebrate subretinal space. Although our experience to date is limited to *Xenopus laevis*, given the similarity of their retinas, the method should be applicable to *Xenopus tropicalis*. The fact that development occurs in the extrauterine environment is an obvious advantage of amphibians over rodents for this method. Subretinal delivery via brain ventricle injection can probably be applied to other vertebrates such as avian embryos where access to the brain ventricle is feasible. A strategy has been described for injection into the brain ventricular system of zebrafish [32,38]; however, to our knowledge, brain ventricle injection has not been performed at the early developmental stages where labeling of the retina might be expected. Nevertheless, the utility of the method in this species may be limited, as the zebrafish optic cup does not appear to form from a typical optic vesicle involution sequence as in other vertebrates [39].

In summary, the technique described here provides a strategy to introduce molecules into the subretinal space without disturbing eye development. The approach, which takes advantage of the common lumen between the brain ventricle and optic vesicles during development, may provide a practical approach to routinely deliver molecules to the subretinal space without disturbing the normal anatomic relationships between the RPE and retina. This could allow the introduction of molecules, including tagged proteins, antibodies, DNA constructs and morpholino oligonucleotides, for cellular and genetic manipulations. The method can probably be adapted to other experimental systems where the optic vesicle and/or embryonic brain ventricle can be accessed.

## Supplementary Material

Supporting Movie
